# Engineering ofloxacin bioavailability through spray-dried HPMC and xanthan gum-based solid dispersions: enhanced solubility and therapeutic efficacy

**DOI:** 10.1039/d5ra06222e

**Published:** 2026-01-28

**Authors:** Sadia Pervez Lali, Arooj Fatima, Muhammad Sher, Muhammad A. Hussain, Muhammad T. Haseeb, Muhammad Naeem-ul-Hassan, Fahad M. Alhoshani, Bandar Khaled Sendy, Ibrahim A. Shaaban, Azhar Abbas

**Affiliations:** a Institute of Chemistry, University of Sargodha Sargodha 40100 Pakistan msherawan@yahoo.com azhar.ramzan@uos.edu.pk +92 48-3768409 +92 300-6022454; b Centre for Organic Chemistry, School of Chemistry, University of the Punjab Lahore 54590 Pakistan; c College of Pharmacy, University of Sargodha Sargodha 40100 Pakistan; d Advanced Diagnostics & Therapeutics Institute Health Sector, King Abdulaziz City for Science and Technology Riyadh Saudi Arabia; e Institute of Wellness and Preventive Medicine Health Sector, King Abdulaziz City for Science and Technology Riyadh Saudi Arabia; f Department of Chemistry, Faculty of Science, Research Center for Advanced Materials Science (RCAMS), King Khalid University P.O. Box 960 Abha 61421 Saudi Arabia; g Government Ambala Muslim Graduate College Sargodha 40100 Pakistan

## Abstract

This study explores the enhancement of oral bioavailability, dissolution rate, and solubility of weakly water-soluble fluoroquinolone antibiotic, ofloxacin (OFL), by solid dispersion (SD) formulation prepared using the spray drying technique. Hydrophilic polymers; hydroxypropyl methylcellulose (HPMC) and xanthan gum (XNG) were used as carriers. FT-IR spectroscopy indicated hydrogen bonding between OFL and the polymer's backbone. DSC and PXRD analyses revealed a transformation from the crystalline to the amorphous state. SEM images revealed reduced particle size and changed surface morphology, which are favorable for solubility improvement. The *in vitro* drug release studies, performed in simulated gastric conditions (pH 6.8) showed a significant improvement in the drug release, 97.88% and 82.34% for HPMC-based (O–H) and XNG-based (O–X) SDs, respectively, as compared to only 59.2% for unprocessed OFL. Further, *in vivo*, kinetics reported in a validated HPLC-UV method in rabbits showed an impressive *C*_max_ (O–H: 4.33 µg mL^−1^; O–X: 4.12 µg mL^−1^; OFL: 1.8 µg mL^−1^) and  prolonged *t*_1_/_2_ (8 h *vs.* 5 h). Thus demonstrating a significant enhancement in bioavailability in the rabbit model. The SDs produced with HPMC and XNG represent a promising strategy to improve the solubility and *in vivo* performance of OFL, which may translate to improved therapeutic efficacy.

## Abstract 2

Polymeric spray-dried dispersions of ofloxacin enhance solubility and bioavailability for improved therapeutic efficacy.

## Graphical abstract

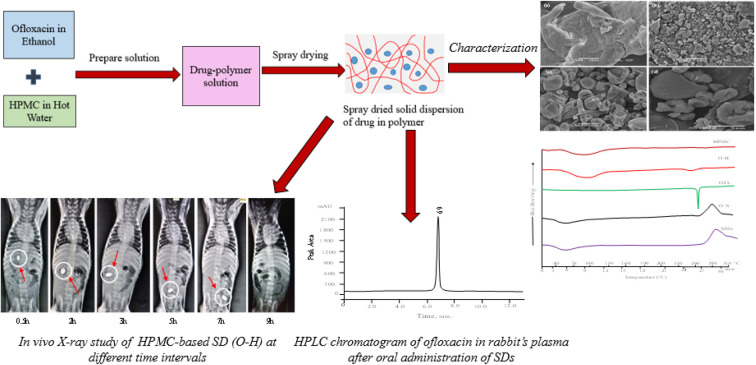

## 1. Introduction

Oral drug delivery continues to be the most commonly used method for administering the therapeutic agents because of its affordability, practicality, and simplicity of use, and better patient compliance.^1^ However, this route is often confronted with the reduced aqueous solubility of most of the recently approved drugs, especially those with Biopharmaceutics Classification System (BCS) Class II and IV. The rate limiting steps include the solubility and dissolution rate in the absorption of the drug and systemic bioavailability for such compounds. Approximately 40% of the pharmaceuticals are marketed drugs, and 90% of drug candidates have solubility-related deficiencies. Therefore, solubility enhancement has become a cogent area of focus in pharmaceutical formulation design.^2–4^

A drug achieves therapeutic efficacy only when its plasma concentration exceeds the minimum effective concentration (MEC) for a specific time. Reaching this point requires the drug to be adequately dissolved in the gastrointestinal (GI) environment and subsequently absorbed into the systemic circulation. Orally administered drugs, as poorly soluble drugs often demonstrate erratic absorption profiles, poor and variable bioavailability, prolonged onset of action and high inter-individual variability. As a result, patients may need higher or more frequent dosing, heightening the risk of adverse effects and reducing the adherence to therapy.^5,6^

Ofloxacin (OFL), a second-generation synthetic fluoroquinolone antibiotic, works against both Gram-positive and Gram-negative bacteria over a wide spectrum. It blocks bacterial DNA gyrase and topoisomerase IV, key enzymes for the replication and proliferation of bacteria. Despite its wide antimicrobial action and its clinical relevance, OFL is only weakly soluble in aqueous environments (2.66 mg mL^−1^ at pH 7 [USP]), limiting its solubility in the GI tract and systemic absorption and therefore, reducing its therapeutic efficacy.^7^ This pharmacokinetic inadequacy has led to investigating the novel formulation approaches to enhance its solubility and bioavailability.^8–10^ Among different solubility enhancement approaches, including micronization, complexation, nanoemulsions and lipid-based delivery systems, solid dispersion (SD) technology is one of the simplest, most economical and applicable to various compound classes. The SDs consist of weakly soluble drugs dispersed within an inert, aqueous polymer matrix, resulting in improved wettability, reduction in crystallinity, increased surface area, and conversion of the drug into a thermodynamically less stable but highly soluble amorphous form.^11^ This modifies effectively the drug solubility and bioavailability.^12^ In addition to traditional supports for SD, the progresses in material science recently even make possible the creative design of multifunctional systems (like fluorescence-sensing MIMs for targeted molecule detection and deep eutectic solvents for green extraction of biopolymers) inspiring construction of more smart responsive delivery platforms.^13,14^ Out of which, the incorporation of nanocomposite catalysts and polyphenol-derived nanomedicines represent the emerging paradigm in multifunctional and biocompatible materials which not only improve solubility but also therapeutic targeting and stability, a concept that defines our choice for HPMC and xanthan gum as advanced polymeric carriers.^15,16^

Other recent SD technology advancements have involved novel polymeric carriers, scalable production processes (*e.g.*, hot-melt extrusion and spray drying) and combination strategies that target solubility and stability. In particular, spray drying is favoured for its potential to generate homogeneous, amorphous dispersions with tuneable particle sizes and morphologies.^17^ One such factor is the choice of polymer that is best suitable for use in SDs. Hydrophilic polymers featuring hydroxypropyl methylcellulose (HPMC)—a semi-synthetic cellulose derivative^18^ and xanthan gum (XNG),^19^ a natural polysaccharide,^20^ have displayed promising compatibilities with multiple active pharmaceutical ingredients (APIs) and can act simultaneously as solubilizers and stabilizers in SD formulations.^21^

Although earlier studies have shown the viability of OFL SDs with carriers such as PEG-6000, little effort has been invested in the application of more versatile, amorphous polymers with added functional value (*i.e.*, controlled release, increased stability) through scalable methods such as spray-drying. This study aims to address this gap by investigating HPMC and xanthan gum as advanced carrier systems. A mini spray-drying procedure incorporates OFL into SDs with HPMC and XNG as polymeric carriers.^22^ These polymers were chosen due to their known safety profiles, biocompatibility, and functional diversity to enhance the drug solubility and delayed drug release. Differential Scanning Calorimetry (DSC), Fourier Transform Infrared Spectroscopy (FT-IR), Powder X-ray Diffraction (PXRD),^23^ and Scanning Electron Microscopy (SEM) were used for the comprehensive characterization of SD formulations to study their molecular interactions, thermal behaviour, physical state, and particle morphology.^24,25^

“While prior research has demonstrated the viability of OFL solid dispersions using carriers like PEG-6000, these systems often rely on semi-crystalline polymers or laboratory-scale methods such as solvent evaporation, which may offer limited physical stability and scalability. This study uniquely addresses this gap by employing the fully amorphous, multifunctional polymers HPMC and xanthan gum, processed *via* the industrially scalable spray-drying technique. Unlike traditional PEG- or PVP-based systems, our approach leverages the superior amorphous-stabilizing capacity of HPMC and the inherent mucoadhesive properties of XNG, which we hypothesize will not only enhance solubility but also prolong gastrointestinal residence time and improve long-term stability. Furthermore, this work provides a comprehensive evaluation beyond dissolution, including detailed *in vivo* pharmacokinetics, haemocompatibility, and real-time GI transit visualization—aspects often lacking in earlier studies—thereby presenting a more advanced and translationally relevant formulation strategy.”

The *in vitro* dissolution was carried out in phosphate buffer (pH 6.8) and the pharmacokinetic (PK) study was conducted *in vivo*, on the rabbits and compared the PK parameters of the drug to determine bioavailability enhancement.^26^ Some haemocompatibility assays were also performed to examine the safety of the formulations for systemic administration. SD tablets were radiographically evaluated in dogs *in vivo* for GI transit behaviour and disintegration patterns. The formulations were finally compared to standard OFL for their ability to inhibit *Staphylococcus aureus* and *Escherichia coli*. This research is intended to present the spray-drying preparation of SDs of OFL, using HPMC and XNG, as a method to improve the solubility issues associated with OFL for enhanced PK profiles and patient compliance, leading to favourable outcomes in the clinical applications.

## 2. Materials and methods

### 2.1. Materials

OFL was obtained from Axis Pharmaceuticals, Faisalabad, Pakistan. HPMC, XNG, potassium dihydrogen phosphate (KH_2_PO_4_), microcrystalline cellulose (MCC), sodium hydroxide (NaOH), analytical grade glacial acetic acid, and absolute ethanol were acquired from Sigma Aldrich, Germany. All the solvents and chemicals were utilized without additional purification and were of analytical quality. This study made use of deionized water.

### 2.2. Preparation of spray-dried solid dispersions

A preliminary study of multiple OFL SD mixes of a wide range of drug-to-polymer ratios allowed us to find the optimum mix proportion that provide the highest solubility, which was found to be 1 : 2. Each batch was prepared as; 5.0 g OFL was added to 250 mL pure ethanol, and 10.0 g polymer (HPMC or XNG) added to 500 mL of piping hot water (40–45 °C) followed by mixing the two and stirring for 2 h. The Spray-drying procedure was carried out at 130 °C inlet temperature, 600 L h^−1^ airflow rate, 4 bar pressure, 38% aspirator rate, 10 mL min^−1^ feed rate were clear solutions of Pilotech YC-015 spray-dried. The SDs were prepared, sieved, and stored in airtight form using a desiccator (the composition is provided in Table S1, SM).

### 2.3. Spectroscopic estimation of OFL in SDs

Spectroscopy method analyzed the concentration of aqueous solution ofloxacin (OFL). The calibration curve was developed using OFL concentrations along with 287 nm absorbance data as the maximum absorbance wavelength value. The quantitative analysis of fluvoxamine within this 2–60 µg mL^−1^ concentration scope proved satisfactory and feasible through Beer–Lambert's linearity parameters. The measurement data showed a confirmed strong linear relationship because the obtained correlation coefficient achieved *R*^2^ = 0.977. The SDs (100 mg) were dissolved in 5% ethanol, agitated for 24 h, filtered and diluted followed by measuring OFL at 287 nm on a UV-Vis spectrophotometer (UV-1700, Shimadzu, Japan). OFL content (%) was determined according to the following equation.1
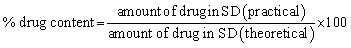


### 2.4. Saturation solubility study

The shake flask method was used to determine the modified water solubility of SDs by dissolving the SDs in ethanol and diluting with water and then shaken at 200 rpm over 24 h using RTSK-0300, Robus Technologies, UK.^27^ The absorbance was recorded at 287 nm with the aid of a UV-Vis spectrophotometer, taking drug-free polymer as blank, and all the tests were performed in triplicate.

### 2.5. Characterization of SDs

FTIR spectroscopy (IR-Prestige 21, Shimadzu, Japan) was employed during the solid-state characterization of OFL, polymers (HPMC and XNG) and prepared SDs (4000–400 cm^−1^), of pelletized samples (KBr), to determine the drug–polymer interactions and then processing the data. Thermal behaviour was determined by DSC (SDT Q 600, TA Instruments, USA), between 25 °C and 310 °C at 10°C min^−1^, under nitrogen and calibrated with indium.^28^ Crystallinity was studied with PXRD (Shimadzu XD-D1, Japan) using Cu-Kalpha aid 50 kV, 40 mA, monitoring the range 10 to 80 (2-theta). Gold-coated sample morphology in SEM (JEOL JSM-5910) at 30 kV 0.25 Torr vacuum was also employed.^28^

### 2.6. Micromeritics studies

The SDs were assessed in terms of their flow and compressibility characters by performing micromeritics studies (Section S1, solid dispersion formulation and physicochemical characterization, of the SI). The angle of repose, mentioned in eqn (1), the bulk and tapped densities are mentioned in eqn (2) and (3), respectively, whilst the Hausner ratio and Carr index are computed through eqn (4) and (5), respectively (SM, S1.1.1–S1.1.5). These parameters offer important data in regards to pre-compression behaviour of the formulation materials (Table S2).

### 2.7. Tablet formation

A combination of standard OFL, corresponding to 100 mg and pure OFL with MCC was accurately mixed and generated a dry mixture. The wet mass production followed and the dry powder mixture was mixed with hot aqueous starch that was heated to 80 °C, resulting in a wet product. The wet mixture underwent a drying process at 80 °C and mesh no. 20 deployed to obtain granular materials. Adding magnesium stearate provided an enhanced flow capacity to the granules. The machine used in compressing the granules was a 7 mm round flat surface punch on ZP-19. Table 1 shows the contents of each tablet.

**Table 1:** Composition of compressed tablets of SDs and OFL

Tablet ingredients	Quantity per tablet		
	O–H	O–X	OFL (standard)
SDs powder (mg)	300	300	—
Standard OFL (mg)	—	—	100
Microcrystalline cellulose (mg)	14	14	214
Starch (mg)	15	15	15
Magnesium stearate (mg)	6	6	6
Total weight (mg)	335	335	335

### 2.8. Post-compression study

The physical and mechanical characteristics of the tablets, subjected to compression, were also examined by means of post-compression analysis in order to assess the hardness, the thickness of the tablets, weight variability, and friability. The gaining and loss of weight were determined according to the formulas (6) and (7), respectively (SM, S1.2.1–S1.2.4). These parameters play a significant role in terms of assuring the uniformity, longevity as well as the quality of final dosage forms. For each batch of formulations, twenty tablets were randomly selected to determine the average weight.^29^

### 2.9. *In vitro* dissolution study

A comparative *in vitro* dissolution test was conducted to determine the profile of drug release parameters of the prepared SDs with that of standard formulation. Dissolution testing was performed on the USP type II apparatus, under given terms, and the analyses were obtained through spectrophotometer at 287 nm (Section S3, biological and performance evaluation, of SM, S3.1). The release behaviour and performance of the formulations, as well as the vital insights are given by this evaluation.

### 2.10. High-performance liquid chromatographic analysis

Quantitative determination and validation of the SD formulations were carried out by high-performance liquid chromatographic (HPLC) analysis. The chromatographic conditions, standard and plasma sample preparation, and the method validation, which made this analytical procedure accurate, precise, and reliable are all given in the Section S2, development and testing of analysis, of SM, S2.1 (S2.1.1–2.1.4).^30^

### 2.11. *In vivo* study design

The PK data comprising *C*_max_ and *T*_max_ were calculated and the area under the curve was calculated from eqn. 8–12 to obtain values of AUCr-t and AUCr-infinity and Cl were calculated using time concentration profile (eqn 8–12). Fifteen rabbits were used in the study, randomly grouped (*n* = 5). The rabbits used in the analysis of the samples were blinded to group assignments. The sample size was selected based on the prior pharmacokinetic research of fluoroquinolones in rabbits, which has also demonstrated consistent results using the same sample size and was also in line with the principles of reduction in animal research but also the sample size was adequate to identify significant differences in the important parameters, such as *C*_max_ and AUC. The detailed dose information per group is given in Table S6, and the outcome in regard to the PK values is given in Table S7, where the bioavailability has been found higher again in SDs than with the pure drug.

### 2.12. Haemocompatibility studies

By assessing the haemocompatibility of SDs in compliance with ISO 10993-4:2017 guidelines, as described in the process provided in Section S3.3 of SM,^26,27^ the risks related to material–blood interactions were quantified. The SDs were incubated with citrate blood and CaCl_2_ in order to determine thrombogenicity gravimetrically (eqn. 13, SM). Each assay contained a negative control (PBS alone) and a positive control (citrate blood with CaCl_2_ but no test material). The formed clots were fixed, dried, and weighed following a 45-minute incubation period at 37 ± 0.5 °C. The thrombose percentage was calculated by normalizing the clot mass of the test sample against the positive and negative controls (eqn. 13, SM). Haemolytic potential was assayed according to the ASTM standard (eqn. 14, SM), wherein SDs were incubated with citrate blood for 3 hours at 37 ± 0.5 °C, then centrifuged, and the haemoglobin release in the supernatant was measured spectrometrically at 540 nm ^30^. Distilled water and PBS served as the positive and negative controls, respectively. The hemolytic index was calculated by normalizing the optical density of the test sample against the controls (eqn. 14, SM). Furthermore, the specific instructions in Sections S3.3.1 and S3.3.2 of the SI have been reinforced to specifically state: The use of a positive control (citrate blood + CaCl_2_) and a negative control (PBS only) for the thrombogenicity test. The exact incubation time of 45 minutes for thrombogenicity. For the hemolysis test, distilled water (positive control) and PBS (negative control) are used, and the incubation period is set at three hours. The hemolytic index (%) and thrombose (%) formulas, which naturally characterize the data normalization procedure against the controls.

### 2.13. *In vivo* X-ray study of SDs

SD tablet gastrointestinal transit in dogs was studied following the procedure given in Section S3.4 of SM. Tablet production consisted of direct tablet pressing of XNG- and HPMC-based SDs using a contrast agent such as 25% BaSO4 to provide the radiographic contrast (Table S2, SM). Study design involved the gavage of O–X and O–H tablets in fasted dogs and serial X-ray imaging at predetermined countenances by using Toshiba radiography equipment to check the location and disintegration of the tablets.^31–34^

### 2.14. *In vitro* antibacterial study

The method of agar well diffusion method was followed in the determination of antibacterial activity against *E. coli* and *S. aureus* of SD formulations as addressed in SM, Sections S3.5.^35^ Measurements of the (inhibition zones) ZoI were carried out using the SD samples to inoculate agar wells containing the test strain and a control, were incubated at 37 °C and the V method was utilized to report the ZoI in millimeters (Tables S13 14, SM). The findings revealed that O–H and O–X had a slightly less ZoI as the standard drug.^35^

### 2.15. Statistical analysis

All the data of *in vitro, in vivo*, and solubility studies were statistically evaluated with GraphPad InStat Software 5 and GraphPad Prism 5.0 (GraphPad Software, USA). The primary method was one-way analysis of variance (ANOVA) and in the areas where it was applicable, Dunnett multiple comparison test was performed. The results were presented as means and SD and the differences were taken to be significant at *p* < 0.05.

## 3. Results and discussion

### 3.1. Spectroscopic estimation of OFL

The optimum concentration of OFL in aqueous media was established spectrophotometrically at 287 nm (after calibrating 2 to 60 g mL^−1^) with intense linearity (*R*^2^ = 0.977), in connection to the Beer–Lambert law. Free OFL had a water solubility of 118.31 ± 0.61 µg mL^−1^ that was taken as a benchmark to compare the solubility improvement following the SDs. The latter ensured the appropriateness of this method regarding quantitative OFL analysis.

### 3.2. Drug content and saturation solubility study

By examining the saturation solubility, optimized SDs O–H and O–X solubilized to 313.21 ± 0.73 and 293.64 ± 0.95, which is approximately 2.5- and 2-fold greater than that of pure OFL (118.31 ± 0.61) (Fig. S1). This was credited to an increase in wettability and minimal recrystallization through using the hydrophilic carriers, where FTIR, DSC and PXRD demonstrated the retention of amorphous drug in the polymer matrix. Analysis of drug content was also very accurate, given as 99.26 lOx 86.33% (O–H), and 98.44 lOx 86.54% (O–X). Saturation solubility and drug contents of OFL, O–H and O–X are presented in Table S9. Such results indicate SDs as a competent method to increase the solubility and clinical efficacy of poorly water-soluble compounds.

### 3.3. Characterization of SDs

#### 3.3.1. Fourier transform infrared spectroscopy (FTIR) analysis

To examine the molecular–level interactions between OFL and polymer carriers HPMC and XNG (Table S3), FTIR analysis was carried out. (SM, Section S1.3). Particular peaks were observed in Pure OFL regarding OH/NH (3400 cm^−1^), C=O (1720 cm^−1^), C=N (1620 cm^−1^), CF (1407 cm^−1^) and C–O–C (1060 cm^−1^). Polymer carriers gave peaks attributable to hydroxyl, ether, and carboxylate. Physical mixtures exhibited overlapping peaks with little shifts suggesting the weak interactions. SDs showed shifts in the OH/NH bands followed by broadening and the intensity of the CO band was also decreased, which supports interaction and low crystallinity. Such spectral transformations confirmed successful effective integration of OFL in a polymer matrix without chemical incompatibility, which confirmed better solubility and dissolution.

#### 3.3.2. Differential scanning calorimetric (DSC) analysis

**Fig. 1:**
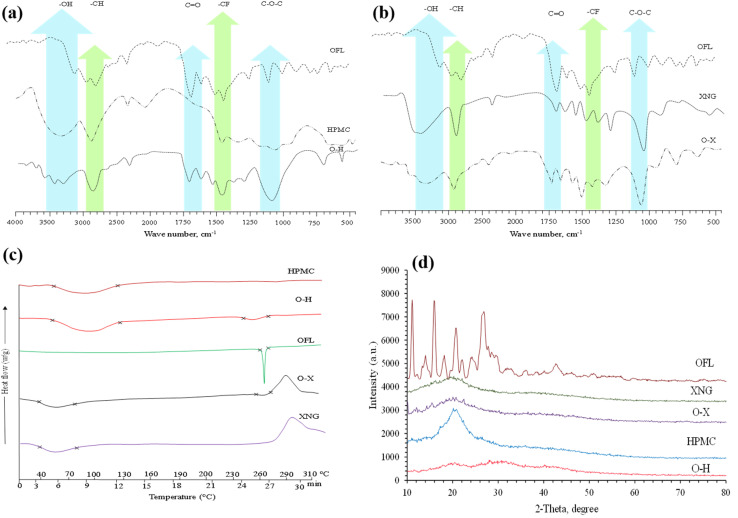
(a) Overlay FT-IR spectra of standard drug (OFL), polymeric carrier (HPMC) and SD (O–H), indicating the presence of all characteristic peaks of the drug and carrier in FT-IR spectrum of SD (O–H), (b) overlay FT-IR spectra of standard drug (OFL), polymeric carrier (HPMC) and SD (O–X), indicating the presence of all characteristic peaks of the drug and carrier in FT-IR spectrum of SD (O–X), (c) overlay DSC thermographs of OFL, HPMC, XNG, O–H and O–X at a heating rate of 10 °C min^−1^ and (d) overlay PXRD patterns of OFL, O–H, O–X, HPMC and XNG, absence of sharp diffraction peaks in PXRD patterns of O–H and O–X, indicating the amorphous nature of SDs.

The DSC analysis of OFL and respective SDs (O–H, O–X) was conducted, and the results are displayed in Fig. 1c and Table S4. The DSC thermogram of OFL showed a single characteristic endothermic peak at 268.2 °C, corresponding to its melting point. The thermogram from HPMC also displayed a wide endothermic peak extending from 62.3 °C to 130.2 °C, which might indicate its *T*_*g*_ as well as dehydration process. Additionally, the characteristic broad peak (40.1–83.9 °C) in the case of XNG also corresponded to the dehydration process.^36,37^ Previous studies have documented a comparable pattern for the DSC curve of XNG.^38^ The disappearance of sharp peaks of OFL in DSC thermograms of O–H and O–X confirms the change of the physical state of OFL (crystalline to non-crystalline) in SDs. The high temperature, pressure, and quick solvent evaporation throughout the spray drying process may be responsible for the phase transition of the drugs in SDs.^39–41^ The results are compatible with the earlier research, where SDs of OFL were developed by utilizing PEG-6000 as a carrier *via* fusion and solvent evaporation method.^42^

#### 3.3.3. Powder X-ray diffraction (PXRD) analysis

The PXRD patterns of OFL, O–H, O–X, HPMC and XNG are presented in Fig. 1d. The diffraction peaks appeared at 11.1°, 14.01°, 15.9°, and 18.14°, 20.7° 26.8° of 2*θ* in the PXRD pattern of OFL, depicted the crystalline nature of OFL. Broad halos in PXRD patterns of polymers at 2*θ* angle of 20.8° (HPMC) and 19.9° (XNG) suggested some orderly arrangements of molecules in amorphous polymers. Moreover, PXRD patterns of O–H and O–X did not exhibit any diffraction peak, which depicted the amorphous physical state of both developed SDs. Hence, the enhanced solubility of SDs, as compared to the standard OFL, may be attributed to its morphological change in structure.

#### 3.3.4. Scanning electron microscopic (SEM) analysis

**Fig. 2:**
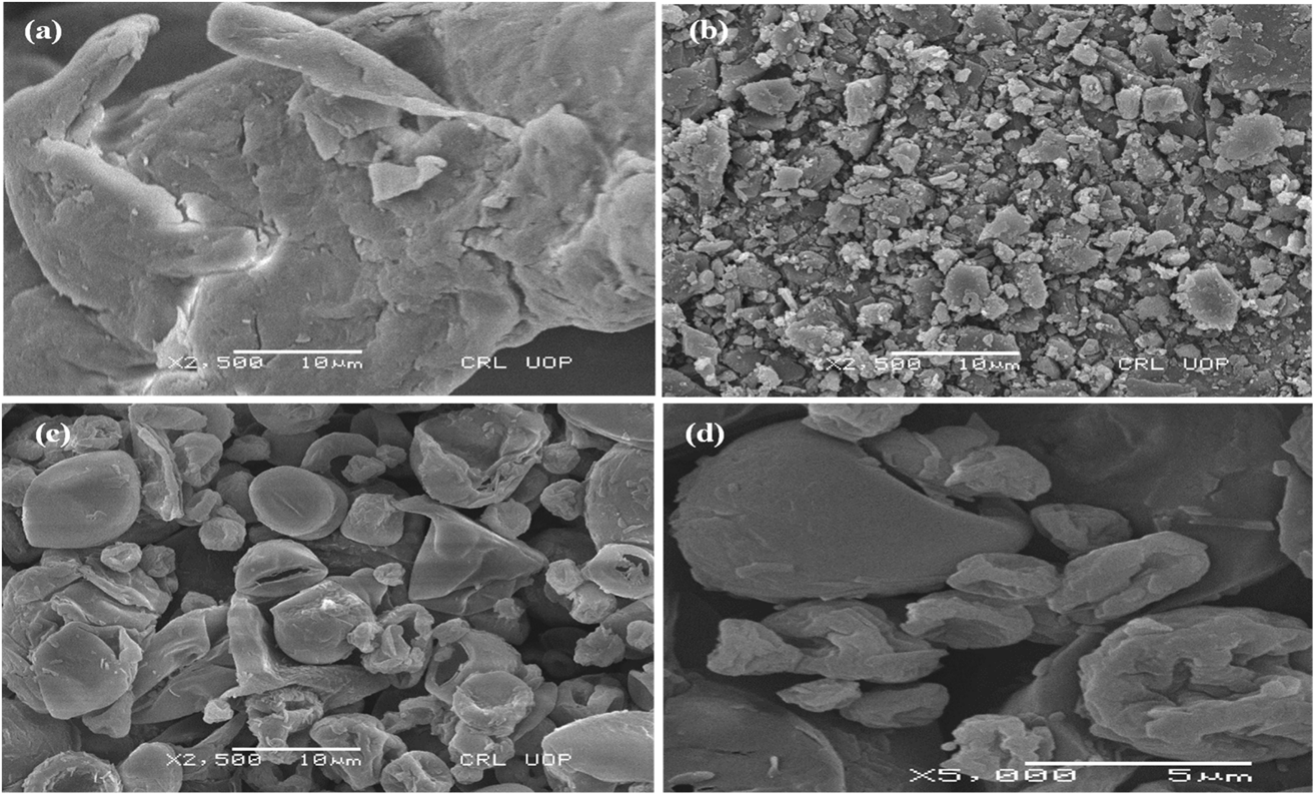
SEM images of HPMC, OFL and O–H (a) SEM image of HPMC, (b) SEM image of OFL, (c) SEM image of O–H and (d) SEM image of O–H at higher magnification level, spherical particles of SD (O–H) having reduced size.

**Fig. 3:**
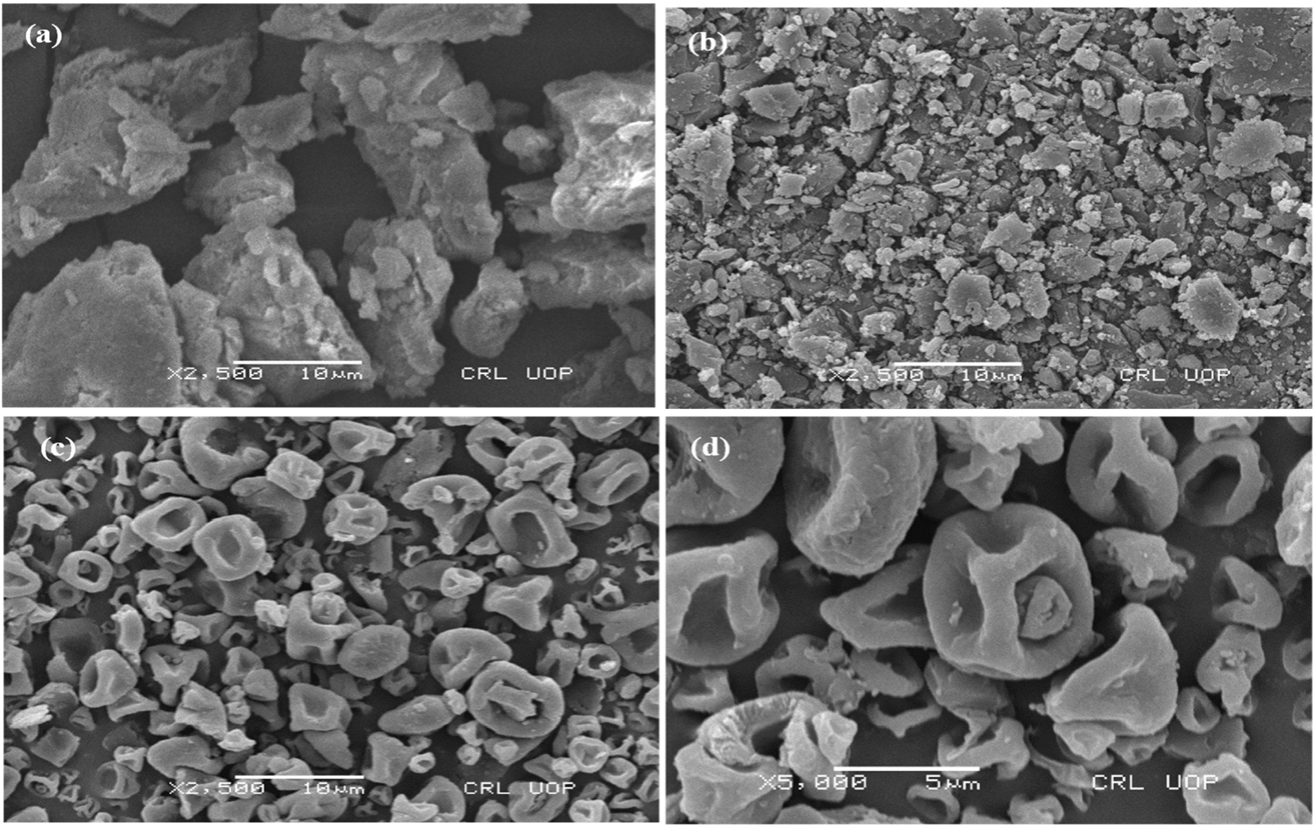
SEM images of XNG, OFL and O–X (a) SEM image of XNG, (b) SEM image of OFL, (c) SEM image of O–X and (d) SEM image of O–X at higher magnification level, spherical particles of SD (O–X) having reduced size.

The SEM image of OFL presented smooth surfaced, long and cylindrical-shaped crystals (Fig. 2b and 3b). Moreover, polymeric carriers (HPMC and XNG) exhibited larger, rougher, and irregular-shaped particles that designated their amorphous morphology (Fig. 2a and 3a). The amorphous nature of O–H and O–X was obvious by the appearance of spherical and irregular particles where remarkably small depressions on the surface above the particles could also be observed, which may be attributed to the quick solvent evaporation throughout the process of spray drying Fig. 2c, d and 3(c, d). Moreover, the disappearance of crystals of OFL concerning O–H and O–X indicated that the drug might be dispersed into the amorphous hydrophilic polymeric carriers.

### 3.4. Physical stability study

The developed SDs underwent accelerated stability tests in accordance with the guidelines of ICH (International Conference on Harmonization). The study was conducted over the duration of six months at 40 ± 5 °C and 75 ± 5% relative humidity (% RH). No momentous variation was observed in the physical appearance and the drug contents of all SDs. The Table S10 shows the percentage drug contents of each formulation after 2, 4 and 6 months.

### 3.5. Rheological properties

Bulk densities and tapped densities of O–H and O–X were 2.61 ± 0.02 and 2.24 ± 0.23 g mL^−1^ and 2.90 ± 0.55 and 2.55 ± 0.03 g mL^−1^ respectively. Values of angles of repose (19.14° + 0.04, O–H; 21.33° + 0.13, O–X) correspond to excellent flow, and Hausner ratios of 1.11 + 0.02 and 1.14 + 0.12 (both, 0.12) and Carr index values of 10.01 + 0.22 and 12.16 + 0.14 percent (both, 18 percent) indicated excellent rheological properties. These data prove an excellent flowability, which is well above pharmacopeial specification. This favourable movement reduces the variation in the weight and content uniformity.^43^ HPMC- and XNG-based SDs achieved the official requirements with regards to the flow properties.

### 3.6. Tablet formation and post-compression study

The hardness and thickness values for compressed SDs tablets (O–H and O–X) were 2.83 ± 0.24, 2.71 ± 0.44 kg cm^−2^ and 2.31 ± 0.42, 2.44 ± 0.71 (mm), respectively, that meets the established criteria. The obtained friability values were 0.83 ± 0.11 and 0.71 ± 0.24 (%), respectively as shown in Table S11. The small friability value of less than 1% confirms the compression tablets have strong mechanical properties that enables the product for typical shipping and packaging stress. The tablet weights exhibited consistent results because the variation values were negligible (2.63 ± 0.43 and 2.73 ± 0.45%) between the batches. Consistent weight of compressed tablets indicates an equivalent particle distribution and uniform sizes. Consistency for all tablet formulations was 99.06 ± 0.34 and 98.12 ± 0.44% for O–H and O–X, respectively (acceptable range, 95 to 105%), showing the uniform distribution of drugs among SD formulations.

### 3.7. *In vitro* dissolution and release kinetics

**Fig. 4:**
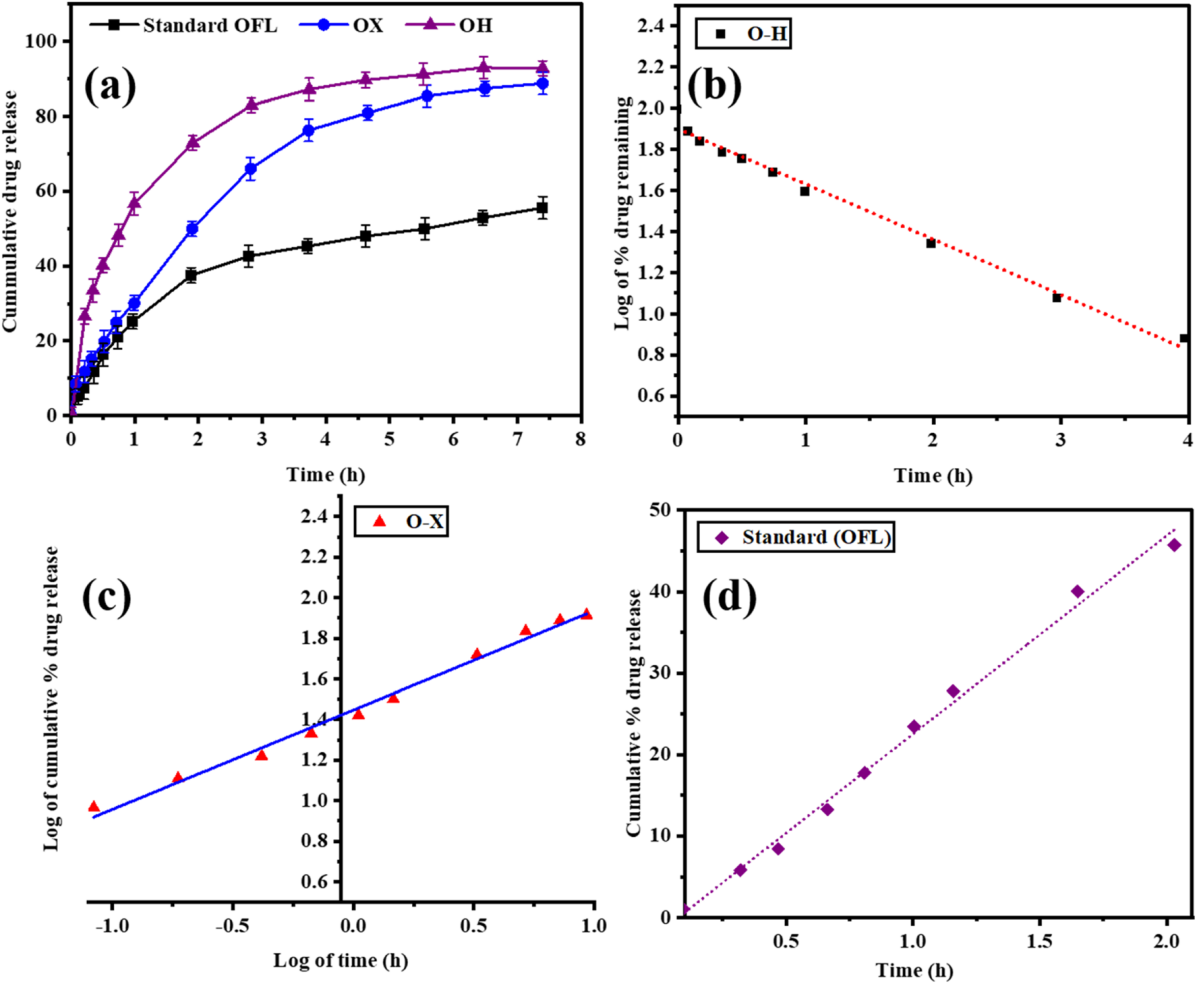
(a) *In vitro* release study of O–H, O–X and standard (OFL) and *In vitro* release kinetics (best-fitted models) of O–H, O–X and standard (OFL) (b) O–H (First order), (c) O–X (Korsmeyer-Peppas model) and (d) OFL (Higuchi model).

Standard OFL and SDs (O–H, O–X) dissolution behaviour was tested in phosphate buffer (pH 6.8) at the temperature of 37 °C (37 °C) ±0.5 °C) over a period of 8 h as outlined in SM, Section S3.5.^44–46^ The *in vitro* dissolution test disclosed maximality of release to reach 59.2, 97.88, and 82.34 percent in case of OFL, O–H and O–X respectively, which was further explained by the hydrophilic character and non-crystalline form of the polymers. Releasing kinetics analysis demonstrated that O–H followed a first-order release, O–X followed the Korsmeyer Peppas model, whereas OFL had mixed erosion/diffusion modes of release and the values of *n* demonstrated that release in the O–H was diffusion-controlled, whereas O–X and OFL were a mixture of erosion and diffusion release models (Table S12, SM) (Fig. 4).

### 3.8. Pharmacokinetic studies

A validated, reverse-phase HPLC/UV method was employed to perform pharmacokinetic analysis of OFL, O–H, and O–X in rabbit models after oral administration of a single dose. Table S5 presents the chromatographic conditions for HPLC analysis of SDs. Validation parameters for HPLC analysis of OFL in plasma are presented in Table 2. The analysis showed a strong correlation coefficient of 0.996 together with a linear range extending from 2 to 60 µg mL^−1^. The data precision in terms of relative standard deviation (% RSD) for three concentrations (2, 12 and 60 µg mL^−1^) was found to be less than 2%, revealing that the proposed method was precise.

**Table 2:** Validation parameters for HPLC analysis of OFL in plasma

Parameters	OFL
Linearity range (µg mL^−1^)	2–60
Regression coefficient (*R*^2^)	0.996
LOD (µg mL^−1^)	2.32
LOQ (µg mL^−1^)	5.14
Precision intra/inter-day (RSD, %)	(1) 0.830/0.342 at 2 µg mL^−1^
	(2) 0.722/0.261 at 12 µg mL^−1^
	(3) 0.931/0.521 at 60 µg mL^−1^
Accuracy (% recovery)	(1) 98.4 at 2 µg mL^−1^
	(2) 96.3 at 12 µg mL^−1^
	(3) 95.2 at 60 µg mL^−1^

**Fig. 5:**
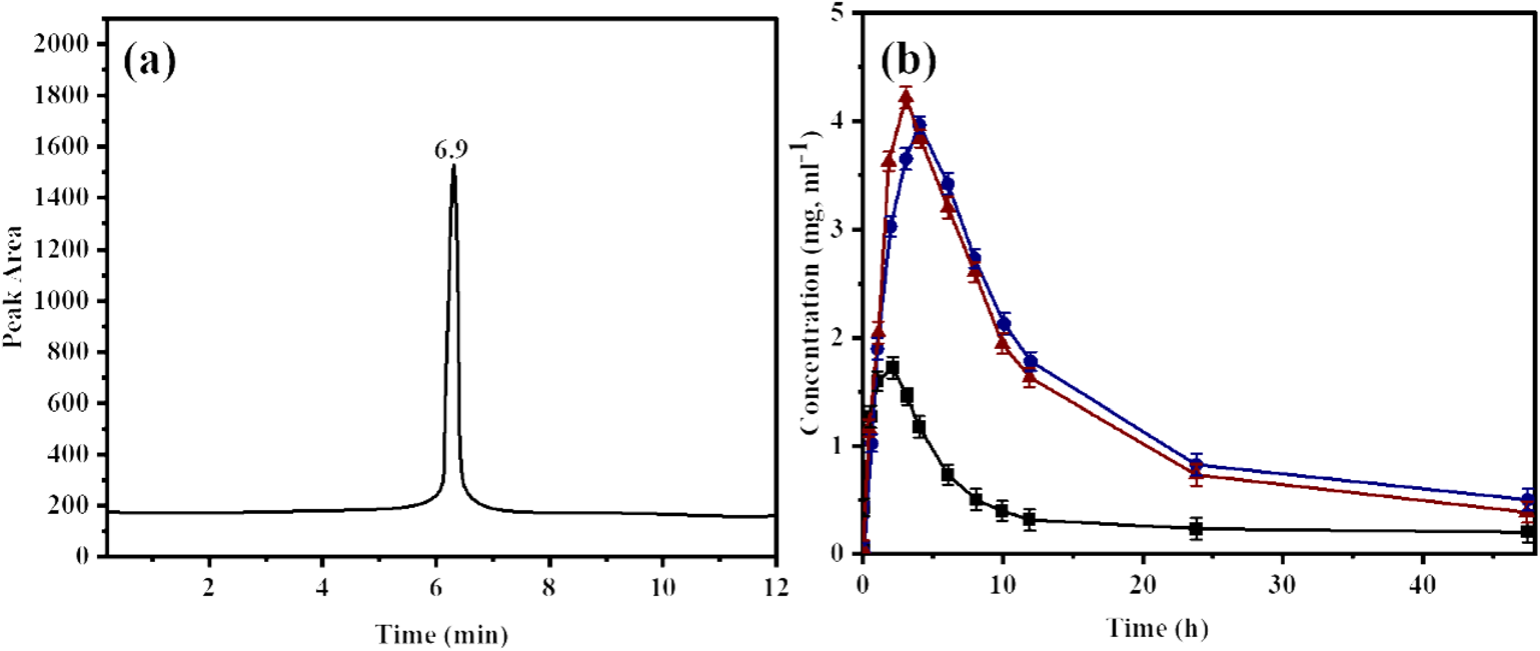
(a) HPLC chromatogram of ofloxacin in rabbit's plasma after oral administration of SDs (O–H and O–X) and (b) overlay plasma concentration *vs.* time curve of O–H, O–X and standard (OFL). Data are presented as mean ± SD. Statistical significance of pharmacokinetic parameters was determined by one-way ANOVA followed by Dunnett's *post hoc* test *p* < 0.05 *vs.* standard OFL.

The accuracy (% recovery) of the developed methods on the same concentration levels was 98.4, 96.3 and 95.2%, respectively. The values of validation parameters revealed the validity of the adopted method. Upon HPLC analysis, the peak for OFL in SDs appeared at 6.98 min., as presented in Fig. 5a.

To study the pharmacokinetic parameters of OFL, O–H, and O–X, their plasma concentrations were determined after administration of single oral dose to rabbits. The plot of plasma concentrations *vs.* time is presented in Fig. 5b. Statistical analysis using one-way ANOVA followed by Dunnett's post-hoc test revealed a marked increase in *C*_max_ values of O–H (4.33 ± 0.21 µg mL^−1^, *p* < 0.01) and O-X (4.12 ± 0.33 µg mL^−1^, *p* < 0.01) as compared to that of standard OFL (1.8 ± 0.61 µg mL^−1^). The increased *C*_max_ values of SDs indicated the augmented solubility of SDs due to the presence of hydrophilic polymeric carriers (HPMC and XNG). Values of AUC_0 − t_ was also increased for O–H and O–X (26.88 ± 3.81 µg L^−1^, *p* < 0.05 and 25.57 ± 4.43 h µg L^−1^, *p* < 0.05, respectively). The same trend was found for all pharmacokinetic parameters (*t*_max_, *t*_1/2_ and Vd), as depicted in Table S4. The increased bioavailability of ofloxacin in SDs may be due to the presence of hydrophilic polysaccharides, which improves the solubility of drugs owing to the enhanced wettability, affinity for water and dispersibility in polymeric carriers.

### 3.9. Haemocompatibility studies

Thrombogenicity analysis (eqn. 13, SM) of O–H and O–X (Section S3.6) revealed the thrombosis values of 78.49 ± 3.77 and 73.91 ± 4.19, respectively presented in Table S8 of SM, and were found lower than the positive control, indicating they are not thrombogenic. The haemolytic indices (eqn. 14, SM) for O–H and O–X were 2.53 ± 1.09% and 1.46 ± 0.69% respectively and were below the ISO 10993-4:2017 safety limit of 5%, and similar to the literature values.^47^ Such findings support the haemocompatibility and biomedical compatibility of HPMC- and XNG-based SDs.

### 3.10. *In vivo* X-ray study

**Fig. 6:**
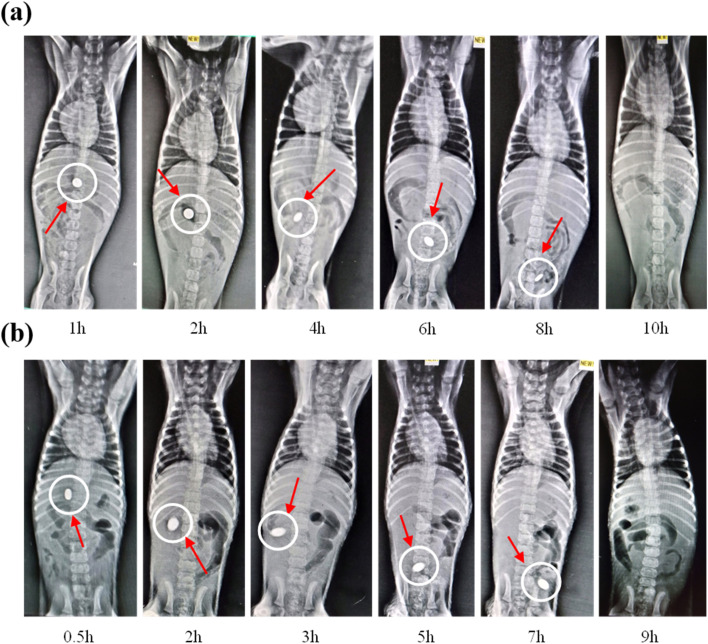
(a) *In vivo* X-ray study of XNG-based SD (O-X) at different time intervals and (b) i*n vivo* X-ray study of HPMC-based SD (O–H) at different time intervals.

The GI transit of O–X tablets was observed to be intact in the stomach after 1 h, then moved slowly through the small bowel at 2 h – 6 h time, and was smaller in the colon at 8 h, and completely disintegrated by 10 h (shown in Section S3.4 and S3.7 of SM). In the O–H tablets, X-ray images showed a smooth movement in the GIT, without causing any serious harm, but they vanished after 9 h. The long-term presence of the tablets showed that the two formulations did not undergo premature disintegration. SDs based on XNG showed a little bit higher residence time in GIT compared to HPMC-based SDs. In general, they both maintained drug release of at least 9 h (Fig. 6).

### 3.11. *In vitro* antibacterial study

The antibacterial activity of SDs against *E. coli* and *S. aureus*, carried out by the disc diffusion method (SM, Section S3.8 (refs. 48 and 49) provided the results (Tables S13 and 14) indicating that the inhibition areas of SDs are similar to the standard OFL. In *E. coli*, O–H and O–X showed zones comparable to the reference (15.5 ± 0.51 mm), whereas against *S. aureus*, the ZoI values were 15.3 ± 1.89 mm (O–H) and 17.1 ± 1.32 mm (O–X) compared with 18.3 ± 0.78 mm of OFL. Negative controls were inactive. The enhanced solubility and dissolution of the SDs likely contribute to their maintained antibacterial effect, which was comparable to the standard drug. This was due to crystalline-to-amorphous transition, decreased particle size, and hydrophilic polymers.^50^ No chemical incompatibility was found, but it was established that there was hydrogen bonding between the drug and carriers. In general, SDs showed better dissolution, oral bioavailability, haemocompatibility, longer GIT residence and antibacterial activity than the conventional drugs.

### 3.12 Going beyond traditional solid dispersion strategies

This research expands on and develops earlier solid dispersion approaches of ofloxacin. As an example, previous research by Pintu *et al.* (2011) was able to increase the rate of dissolution of OFL through PEG-6000 carrier through fusion and solvent evaporation strategies. Our strategy with HPMC and xanthan gum is effective, but it has a number of clear benefits. To begin with, as opposed to the semi-crystalline structure of PEG, both HPMC and XNG are amorphous polymers that have been shown to be capable of enhancing physical stability of the amorphous drug and therefore reduce occurrence of recrystallization and ensure long-term stability as evidenced by our ICH stability studies.^8,38^ Secondly, there is added functionality of these polymers: HPMC is reputed to have gel-forming properties capable of regulating drug release and xanthan gum, being a naturally occurring polysaccharide, provides biocompatibility and possible mucoadhesivity, potentially increasing gastrointestinal residence time, as proposed by our *in vivo* X-ray analyses.^17,51^ Thirdly, we used the spray-drying method, a highly scalable and repeatable industrial procedure relative to laboratory-scale procedures such as fusion or solvent evaporation, making it easier to transition to technology. Lastly, our study has a more thorough evaluation, featuring not only dissolution improvement, but also detailed pharmacokinetic studies in an animal model, haemocompatibility tests, and direct visualization of GI transit, which had been lacking in previous literature (Table 3).

**Table 3:** Comparison of some previously developed SD formulations of fluoroquinolones with the current study

Formulation/strategy	Polymer used	Results/advantage	Reference
Fusion and solvent evaporation strategies	PEG-6000	Increase the rate of dissolution of OFL	42
Amorphous SDs by ball milling the ciprofloxacin with polymers	Eudragit L100, Eudragit L100, carbopol & HPMCAS	Improved solubility, bioavailability and antimicrobial activity	52
Zero-order sustained release mucoadhesive minitablet SDs	Eudragit^®^ RS, carbopol	The formulation exhibited superior microbiological activity than commercial formulations after once daily administration	53
Gastro retentive floating levofloxacin tablets by nonaquous granulation method	HPMC-K4M and Carbopol-940	The formulation was useful for the sustained delivery of levofloxacin to treat peptic ulcer	54
Floating tablets of levofloxacin by direct compression	HPMC, CMC, and starch	Improvement in the gastric residence time and therapeutic outcomes	55
Sustained-release matrix tablet for levofloxacin	Carrageenan (CRG) as a natural, multifunctional excipient	The potential of CRG as an effective matrix-forming polymer in oral sustained-release systems has been established	56
Amorphous solid dispersion of ciprofloxacin for inhalation	Gelatin	Demonstrated the potential of gelatin to enhance the solubility of ciprofloxacin, a poorly soluble drug	57
Swellable and floating gastroretentive drug delivery system for ciprofloxacin	Sangelose^®^ and HPMC	Rapidly achieved the therapeutic concentration and maintained the plasma antibiotic concentration for an extended period	58
Gastroretentive floating drug delivery system of levofloxacin	Alovera hydrogel and cellulose	The formulation remained buoyant (>12 h) in the simulated gastric fluid with a buoyancy time of 303 s	59
Pharmaceutical and pharmacological evaluation of amoxicillin	Β-Cyclodextrin and HPMC	Enhanced sustained release was achieved	60
Solid dispersion formulation of ofloxacin prepared using the spray drying technique	HPMC and xanthan gum	Enhanced the solubility and an improved bioavailability has been observed in rabbit models	This study

Thus, the current system is a major improvement as it incorporates a scalable manufacturing process and a multifunctional polymeric carrier to establish a stable, safe and bioavailable formulation of OFL.

## 4. Conclusion

In the present study, Ofloxacin antibiotic based SD formulations were developed through a spray drying technique with hydrophilic polymers, which included HPMC and XNG. The enhanced solubility was mainly a result of three significant aspects: the conversion of the drug from crystalline to amorphous form, particle size reduction, and the hydrophilic character of the polymeric carriers. The prepared SDs and powders exhibited good pre- and post-compression characteristics, such as acceptable flow properties, enhanced mechanical strength, and uniformity in drug content. The *in vitro* dissolution study showed a significantly increased drug release rate, suggesting the potentially improved oral bioavailability. SDs also demonstrated both, the haemocompatibility and significant antibacterial efficacy against different strains of Gram-positive and Gram-negative bacteria. *In vivo* X-ray imaging showed that the tablets remained unbroken and degraded within the digestive system over time, further suggesting their promise for controlled release applications. The results of this study are promising and demonstrate the potential of these SD formulations. However, to confirm these findings for human application, further *in vivo* studies in human subjects are necessary. In addition, extensive toxicity studies, including acute, sub-acute, and chronic studies, must be followed to confirm safety in the long run before utilizing these novel drug delivery systems in clinical settings.

## Author contributions

Sadia Parvez Lali: methodology; investigation; writing – original draft. Arooj Fatima: writing – review & editing. Muhammad Sher: conceptualization; supervision; funding acquisition; validation; project administration; writing – review & editing. Muhammad A. Hussain: supervision; validation; writing – review & editing. Muhammad T. Haseeb: writing – review & editing. Muhammad Naeem-ul-Hassan: writing – review & editing. Fahad M. Alhoshani: writing – review & editing. Bandar Khaled Sendy: writing – review & editing. Ibrahim A. Shaaban: validation; writing – review & editing.Azhar Abbas: validation; writing – review & editing.

## Conflicts of interest

The authors declare that they have no known competing financial interests or personal relationships that could have appeared to influence the work reported in this paper.

## Supplementary Material

RA-016-D5RA06222E-s001

## Data Availability

The data supporting this article have been included as part of the Supplementary Information. Supplementary information (SI) is available. See DOI: https://doi.org/10.1039/d5ra06222e.
