# Supplementary material for: Engineering ofloxacin bioavailability through spray-dried HPMC and xanthan gum-based solid dispersions: enhanced solubility and therapeutic efficacy
Source: RSC Adv. 2026 Jan 28;16(7):6048–60. doi: 10.1039/d5ra06222e (PMC12848879; doi:10.1039/d5ra06222e)
Supplement: RA-016-D5RA06222E-s001 [file RA-016-D5RA06222E-s001.pdf]

Supplementary Information (SI) for RSC Advances.
This journal is © The Royal Society of Chemistry 2026

Engineering Ofloxacin Bioavailability through Spray-Dried HPMC and Xanthan Gum-Based Solid Dispersions: Enhanced Solubility and Therapeutic Efficacy

Sadia Pervez Lali [a], Arooj Fatima [a], Muhammad Sher [a,], Muhammad A. Hussain [b], Muhammad T. Haseeb [c], Muhammad Naeem-ul-Hassan [a], Fahad N. Alhoshani[d], Bandar Khaled Sendy[e], Ibrahim A. Shaaban[f], Azhar Abbas [a, g]**

[a] *Institute of Chemistry, University of Sargodha, Sargodha 40100, Pakistan*

[b] *Centre for Organic Chemistry, School of Chemistry, University of the Punjab, Lahore 54590, Pakistan*

[c] *College of Pharmacy, University of Sargodha, Sargodha 40100, Pakistan*

[d]*Advanced Diagnostics & Therapeutics Institute Health Sector, King Abdulaziz City for Science and Technology, Riyadh, Saudi Arabia*

[e]*Institute of Wellness and Preventive Medicine Health Sector, King Abdulaziz City for Science and Technology, Riyadh, Saudi Arabiaf*

[f]*Department of Chemistry, Faculty of Science, Research Center for Advanced Materials Science (RCAMS), King Khalid University, P.O. Box 960, Abha, 61421, Saudi Arabi*

[g] *Government Ambala Muslim Graduate College, Sargodha 40100, Pakistan*

** Corresponding authors:*

Prof. Muhammad Sher

Mailing address: Ibne-Sina Block, Institute of Chemistry, University of Sargodha, Sargodha 40100, Pakistan. Phone: (0092) 300-6022454. Fax: (0092) 48-3768409
E-mail: msherawan@yahoo.com

Dr Azhar Abbas

Govt. Ambala Muslim Graduate College, Sargodha, 40100, Pakistan; https://orcid.org/0000-0002-5741-7136*: Email:* azhar.ramzan@uos.edu.pk

Contents:

S1. Solid Dispersion Formulation and Physicochemical Characterization

S1.1 Micromeritics studies

S1.1.1 Angle of repose

S1.1.2. Bulk density

S1.1.3. Tapped density

S1.1.4. Hausner's ratio

S1.1.5. Carr's index

S1.2. Post-compression study

S1.2.1. Hardness

S1.2.2. Thickness and diameter

S1.2.3. Weight variation

S1.2.4. Friability test

S1.3. Fourier transform infrared spectroscopy (FTIR) analysis

S2. Development and testing of analysis

S2.1. High-performance liquid chromatographic analysis

S2.1.1. Chromatographic conditions

S2.1.2. Preparation of standard solutions of drugs and samples (SDs)

S2.1.3. Preparation of blank plasma solution

S2.1.4. Analytical method validation

S3. Biological and Performance Evaluation.

S3.1. In vitro dissolution study

S3.2. In vivo study design

S3.2.1. Specimen collection and storage

S3.2.2. Preparation of plasma sample

S3.2.3. Pharmacokinetic parameters

S3.3. Haemocompatibility studies

S3.3.1. Thrombogenicity evaluation

S3.3.2. Haemolytic potential

S3.4. In vivo X-ray study of SDs
S3.4.1. Preparation of tablets
S3.4.2. Study design
S3.5. In vitro dissolution and release kinetics
S3.6. Haemocompatibility studies
S3.7. In vivo X-ray study
S3.8. In vitro antibacterial study

S1. Solid Dispersion Formulation and Physicochemical Characterization

S1.1 Micromeritics studies

S1.1.1 Angle of repose

The fixed funnel method was used to evaluate the angle of repose for all developed SDs. A fixed funnel positioning at 2 cm above a flat surface allowed the measurement of formed pile diameter with graph paper beneath it. The funnel operation lasted until the upper edge of the pile reached the lower edge of the funnel tip. The analysts documented the diameter and height of each pile base to determine pile angles through Equation 2.

$$\theta = tan - 1 \, {}^{h}/_{r} \tag{1}$$

where θ represents the angle of repose, r designates the radius, and h stands for the height of the heap.

S1.1.2. Bulk density

Bulk volume of each powder SD was measured after accurate weight measurement into the 10 mL measuring cylinder. Evaluation of bulk density required the use of Equation 3.

$$D_b = \frac{M}{V_b} \tag{2}$$

where D_b represents the bulk density, M denotes the mass, and V_b denotes the bulk volume of the powder SDs.

S1.1.3. Tapped density

The measuring cylinder needed to be tapped for reaching its constant volume termed as the tapped volume for following calculations. The calculation method for tapped density measurement is represented by the expression in (4).

$$D_t = \frac{M}{V_t} \quad (3)$$

where D_t, M, and V_t are the tapped density, mass, and tapped volume of SD powder, respectively.

S1.1.4. Hausner's ratio

Hausner ratios was calculated through the division of tapped density data by bulk density values according to Equation 5.

$$\textit{Hausner's ratio} = \frac{\textit{Tapped density}}{\textit{bulk density}} \quad (4)$$

S1.1.5. Carr's index

The measurement of material compressibility depends on the calculation of Carr's index. Equation 6 with measured values of bulk and tapped density enabled the calculation of Carr's index.

$$\textit{Carr's index (\%)} = \frac{\textit{Tapped density} - \textit{Bulk density}}{\textit{Tapped density}} \times 100 \quad (5)$$

S1.2. Post-compression study

S1.2.1. Hardness

The digital tablet tester PTB 311(511)-E from Pharma Test German confirmed the hardness levels of twenty randomly chosen tablets for each prepared formulation. The assessment of tablet hardness levels took place at regular time points after tablet breakdown. The calculation occurred to determine the mean values with standard deviation.

S1.2.2. Thickness and diameter

The randomly selected tablets from each formulation received vertical placement between the digital tablet tester arms (PTB 311(511)-E, Pharma Test, Germany). The dimensions of thickness and diameter from the tablet automatically displayed on the digital screen

S1.2.3. Weight variation

A weight variation test determines the weight criteria for tablets to be uniform. The researchers determined average weight after choosing twenty pills at random from each formulation batch. The weights of individually weighed chosen pills helped determine weight variation through use of Equation 7.

$$Weight\ variation\ (\%) = \frac{Wa - Wi}{Wa} \times 100 \quad (6)$$

The weight calculation compares Wa as average weight to the singular weight Wi from each tablet batch.

S1.2.4. Friability test

The measure of solid dosage form tolerance towards surface damage occurs during packing activities and handling and transportation steps is termed friability. The determination of friability occurs through weighing the solid dosage forms before and after particle loss from their surfaces occurs. The tablets should undergo a maximum weight loss of 1% during testing. A weight determination involved ten tablets from each batch before the study commenced (Wi). The selected tablets underwent testing at 25 rpm for four minutes in the automatic device PTF E/ER manufactured by Pharma Test located in Germany. To determine the friability two additional weighings of the previous mentioned tablets were performed by utilizing Equation 8.

$$Friability\ (\%) = \frac{W_i - W_f}{W_f} x\ 100 \quad (7)$$

S1.3. Fourier transform infrared spectroscopy (FTIR) analysis

The FT-IR spectra of the analyzed samples offer important information about the molecular bonding between ofloxacin (OFL) and the polymeric carriers that form the solid dispersion (SD) formulations. Pure OFL exhibited clear peaks in the regions of interest centred at approximately 3400 cm^{-1} for O-H and N-H stretching vibrations, at 1720 cm^{-1} for C=O stretching, and at nearly 1620 cm^{-1} corresponding to the C=N stretching vibrations associated with the quinolone moiety as shown in Fig. 1a. Characteristic C-F stretching and other fingerprint region peaks also developed between 1250 and 1050 cm^{-1}. HPMC hydroxyl groups accounted for broad O-H stretching bands near 3400 cm^{-1} along with C-H stretching 2900 cm^{-1} and C-O-C ether linkages in 1050–1150 cm^{-1} ranges, strong in nature. XNG exhibited peak at 3400 cm^{-1} for O-H stretching, 1600–1650 cm^{-1} for COO^{-} asymmetric, and 1030–1070 cm^{-1} for C-O vibrations.

The spectra for the physical mixtures (PM) revealed the peaks of OFL, as well as those of the polymers, with little shift, indicating only weak physical interaction. The spectra of the solid dispersion showed marked changes. The O-H/N-H stretching broad peak at around 3400 cm^{-1} shifted and strengthened, demonstrating strong hydrogen bonding between the OFL and polymers. Moreover, its carbonyl (C═O) stretching peak at around 1720 cm^{-1} was either reduced or shifted due to interactions with the polymer, and the C═N + C-F peaks of OFL were weakened or diminished in intensity, which indicates that the drug molecule lost its crystalline feature through molecular dispersion or amorphous dispersion in the polymer.

Such spectral changes are significant and indeed confirm molecular level interactions between OFL with either HPMC or XNG in the SD formulations. Additional evidence also came from DSC and PXRD analysis, which was supported by the FT-IR findings, which were in line with the transformation of OFL from crystalline to non crystaline form. The enhancement in solubility and dispersion of OFL can be directly correlated to the improvement of in vitro dissolution and in vivo pharmacokinetics performance, which is done due to the development of hydrogen bonds and reduction of crystalline peaks. In general, the FT-IR analysis confirms the successful preparation of stable and amorphous solid dispersions with enhanced drug delivery prospects.

The FT-IR spectra of pure ofloxacin (OFL), the polymeric carrier xanthan gum (XNG), and the optimized solid dispersion (O-X), are shown in Fig. 1b which illustrates the molecular interactions and structural characteristics. The OFL spectrum displayed peaks at about 3400 cm^{-1} attributed to stretching modes H-O and H-N, peaks around 2900 cm^{-1} due to stretching of C-H, 1720 cm^{-1} related to stretching of C=O, between 1450 and 1300 cm^{-1} peaks attributed the H-F form, and between 1050–1150 cm^{-1} reflecting C-O-C stretching, which confirm the drug functional groups when crystallized. The XNG spectrum also shows a wide O-H band at around 3400 cm^{-1}, characteristic of its polysaccharide nature, and bands in the fingerprint region consistent with its sugar backbone.

The characteristic peaks are still present in the FT-IR spectrum of the solid dispersion (O-X), but there are significant differences. The O-H/ N-H band centred around 3400 cm^{-1} broadens and slightly shifts, a sign of hydrogen bonding formation between OFL and the polymer matrix. The intensity of the C=O stretching peak (1720 cm^{-1}) is decreased, which indicates a lower crystallinity of OFL and its possible participation in the interaction with the carrier groups. The C-F and C-O-C stretching bands with low intensities may suggest that the drug has been molecularly scattered in the polymer and may have undergone some structural reorganization.

These spectral changes confirm that the drug has undergone vigorous intermolecular interactions with the polymers in the process of solid dispersion formation. Moreover, significant variations were found in peak positions and intensities (especially in the hydrogen bonding and carbonyl regions), indicating the successful incorporation of OFL into the polymer matrix on a molecular level, which facilitates drug dispersion and decreased crystallinity. This underpins improving drug solubility and a better-performing formulation. All peaks observed in FT-IR spectra from drug compounds together with polymers also appeared in the respective SDS. Table S7. Indicated that there is no incompatibility between standard OFL and both polymeric carriers. These findings revealed no chemical interaction between drugs and polymers.

Table S1. Composition of prepared SDs of OFL.

Formulation code	Drug	Polymer	Drug: polymer ratio (w/w)	Yield (%)

O-H	Ofloxacin	HPMC	1:2	79.62
O-X	Ofloxacin	XNG	1:2	74.55

Table S2. Pre-compression parameters of SD formulations

SD Formulation	The angle of repose (θ)	Bulk density (g mL^{-1})	Tapped density (g mL^{-1})	Hausner's ratio	Carr's index (%)
O-H	19.14 ± 0.04	2.61 ± 0.02	2.90 ± 0.55	1.11 ± 0.02	10.01 ± 0.22
O-X	21.33 ± 0.13	2.24 ± 0.23	2.55 ± 0.03	1.14 ± 0.12	12.16 ± 0.14

Table S3. FT-IR analysis of OFL, HPMC, XNG, O-H and O-X

Functional group	Characteristic peaks (cm^{-1})				
	OFL	HPMC	XNG	O-H	O-X
-OH	3332	3473	3493	3328	3345
-CH	2918	2906	2897	2908	2910
C=O	1708	-	1720	1712	1710
-CF	1407	-	-	1410	1408
C-O-C	1061	1055	1049	1055	1051

Table S4. DSC analysis of OFL, O-H, O-X, HPMC and XNG

Sample	T_o (°C)	T_E (°C)	T_P (°C)
OFL	263.3	269.5	268.2
HPMC	62.3	130.2	96.6
XNG	40.1	83.9	60.7
	280.1	303.1	295.2
O-H	58.2	128.4	99.9
	247.2	269.6	266.7
O-X	40.2	79.8	61.5
	258.3	272.3	267.9

T_o (Onset temperature), T_E (Endset temperature), T_P (Peak temperature)

S2. Development and testing of analysis

S2.1. High-performance liquid chromatographic analysis

A previously documented HPLC examination method served for the analysis of OFL suspension drops.

S2.1.1. Chromatographic conditions

Product development occurred at the German facility of the Agilent 1200 series HPLC system. The Agilent 1200 series HPLC system contained four major components including the diode array detector (G1315B), autosampler (G1329A), degasser (G1322A) and column oven (G1316A) and quaternary pump (G1311A). The Shim-Pak ODS 5 µm (4.6 × 250 mm) column constituted the analytical system. The procedure required optimal mobile phases to perform HPLC examinations of compounded SDs. Different compositions of selected mobile phase solutions were tested for achieving optimal chromatographic performance when analyzing the produced SDs. The mobile phase selection process involved gradient separations as part of choosing ideal separation conditions. The selected mobile phase identified was used to perform all separations under isocratic conditions. The mobile phases underwent degassing through membrane filtration with a 0.45 µm pore size before their use. The column oven ran at 30°C under conditions of a 20 µL injection volume. Table S12. presents HPLC analysis conditions for the determination of SDs as summarized in this document.

S2.1.2. Preparation of standard solutions of drugs and samples (SDs)

Standard OFL required precise weighing (100 mg) before dissolving it in 100 mL mobile phase to prepare standard stock solutions at 1000 ug/ml concentration. A precise 10 mL stock solution was extended to 100 mL to produce a concentration of 100 µg/mL. The analysis required a degassing step followed by proper filtration through a 0.45µm filter before sample dilution.

S2.1.3. Preparation of blank plasma solution

The blood sample of 4-5 mL from a healthy rabbit male white albino was obtained inside a lithium heparin collection tube that served as an anticoagulant. The blood samples received centrifugation for five minutes under 3500 × g speed. The micropipette equipped with a sucker enabled the removal of the plasma supernatant layer. The plasma samples received storage within test tubes which contained heparin solution and displayed label identification. During HPLC analysis the tubes were placed at -20 °C while wrapped in aluminum foil before completion of the tests. The researcher performed a second round of centrifugation at 3500 × g for 5 minutes following plasma addition of 1 mL acetonitrile and acetic acid each. The syringe contained a 0.45 µm filter which separated and filtered the supernatant layer.

S2.1.4. Analytical method validation

The International Conference on Harmonization standards required analytical method validation to confirm the reliability of the analysis procedure.

To assess accuracy and precision, a series of quality control samples in plasma were meticulously prepared at three distinct concentrations (2, 12, and 60 µg/mL), effectively covering the entire range of the standard curve, which represented low, middle, and high-quality control samples. Each standard solution was measured in triplicate, and the recovery percentage was computed. Subsequently, each concentration solution was subjected to analysis three times under optimal chromatographic conditions, allowing for the determination of mean values and relative standard deviations. Finally, the mean and relative standard deviation were thoroughly calculated. Prior to conducting the assay, it was essential to ensure the equilibration of the chromatographic column by running the mobile phase through the system for at least one hour. Furthermore, system suitability parameters, such as capacity factor, theoretical plates, and resolution, were determined according to the specifications outlined in USP XXIII. For the actual analysis, solutions comprising blanks, standards, and samples (each 20 µL in volume) were injected into the chromatograph, employing the chromatographic conditions previously described. This entire experiment was conducted in triplicate to comprehensively evaluate linearity, sensitivity, accuracy, precision, specificity, and robustness.

Table S5. Chromatographic conditions for HPLC analysis of SDs

SDS	Mobile phase	Ratio (v/v)	Flow rate (mL min^{-1})	Detection wavelength (nm)
O-H	Methanol: Acetonitrile	65: 35	1.5	287
O-X	Methanol: Acetonitrile	65: 35	1.5	287

S3. Biological and Performance Evaluation.

S3.1. *In vitro* dissolution study

All commercialized and developed formulations (standards) used compressed tablets for their in vitro dissolution testing. PTCF 11 Pharma test (Germany) dissolution apparatus type II operated for in vitro release testing of samples that followed predetermined temperatures of 37 ± 0.5 °C and rotational speed of 100 rpm inside a 900 mL pH 6.8 dissolving medium. Throughout the experiment duration of eight hours researchers collected five milliliter aliquots from each sample at predetermined time points. Fresh dissolving media replaced the withdrawn volume in order to maintain stable sink conditions during the experiment. The analysis was performed using Whatman filter paper to filter each solution followed by analysis on a UV/Vis Spectrophotometer (UV-1700 Shimadzu, Japan). The absorbance measurement of OFL took place at a specific wavelength which was 287 nm. The buffer solution used for the blank measurement contained phosphate salts with a pH value of 6.8. The dissolution experiments have been carried out three times and calculated the final value from these trials.

S3.2. *In vivo* study design

The one-way analysis of variance (ANOVA) on solubility and dissolution research data was conducted using GraphPad InStat Software 5 developed by GraphPad Software USA. Research showed differences were statistically important when the p value decreased below 0.05.

S3.2.1. Specimen collection and storage

The researcher obtained three to five milliliters of blood using heparinized disposable syringes from the jugular veins of rabbits both in the experimental and control groups. The subject received

their medication by mouth then scientists collected blood samples at specific time points that started with 0.5 hour and ended at 48.0 hours after drug intake. The blood samples underwent a six-minute spin at 3500 × g while being treated with heparinization and labeling stages. A micropipette equipped with a sucker functioned to extract the supernatant plasma layer. The plasma samples were shifted to heparinized test tubes which were then capped and labeled before the tubes received aluminum foil seals. The HPLC procedure waited for the HPLC test completion while the samples were stored at -10 °C.

S3.2.2. Preparation of plasma sample

To precipitate out proteins from each plasma sample, methanol and acetonitrile (1 mL each) were added to the samples. All plasma samples were centrifuged at 3500 × g, and the clear supernatant was collected with a syringe. The supernatant was filtered through 0.45 μm nylon syringe filters and subjected to HPLC analysis.

S3.2.3. Pharmacokinetic parameters

A concentration-time curve helped determine the plasma concentration measurements for sample and standard medications based on the developed research method. The graph provided values to determine both Tmax and Cmax. The calculation of area under the curve used the following relation to determine from time zero to time t (AUC0-t) and zero to infinity (AUC0-∞) values:

$$AUC_{0-\infty} = AUC_{0-t} + AUC_{t-\infty} \quad (8)$$

AUC0–∞ = AUC0–t + AUCt–∞

and

$$AUC_{t-\infty} = \frac{C_t}{k} \quad (9)$$

AUCt–∞ = Ct/k

The method evaluates drug absorption by calculating Ct at time t using k as the rate constant to determine total drug exposure through AUCt–∞ area under the plasma concentration-time curve from (t) to ∞.

A linear trapezoidal method computed the AUC for all data points preceding the terminal ones in plasma concentration curves. A regression analysis determined the value of rate constant (k) from the last three points on the plasma concentration-time curve. The low slope of the last four data points on the curve represented elimination rate constant (ke). Computations of volume of distribution (Vd) and clearance (Cl) and half-life (t1/2) values were obtained.

$$t_{1/2} = \frac{0.693}{k_e} \qquad (10)$$

$$C_l = \frac{dose}{AUC_{0-\infty}} \qquad (11)$$

$$V_d = \frac{C_l}{k_e} \qquad (12)$$

All recorded values were displayed as their mean numbers with standard deviation (± SD) values represented as well. The statistical research was performed with the help of Statgraphics® 5.1 software.

S3.3. Haemocompatibility studies

The tests checked haemocompatibility to evaluate material-blood incompatibility potential. Standard operating procedures described by ISO 10993-4: 2017 guided the tests of haemolytic potential and thrombogenicity for the material.

S3.3.1. Thrombogenicity evaluation

Thrombogenicity testing of XNG (O-X) and HPMC (O-H) SDs was done by weighing them according to the gravimetric method. The experiment involved weighing each sample precisely to 500 milligrams then submerging them in phosphate buffer saline for the duration of twenty-four hours. The experimental setup maintained the test apparatus at 37 degree Celsius ± 0.5 for the duration of the research.

The researchers discarded the extra PBS solution after 24 hours before adding 2 mL of citrate blood along with 0.2 mL of CaCl2 (0.1 M). The mixture received distilled water at minute 45 to avoid blood clotting. Experimental conditions required formaldehyde at the concentration of 36-38% to fix the clots (5 mL). Laboratory testing involved weighing accurately the dried clots that had been obtained from the SD material to measure their thrombogenic characteristics. The procedure for preparing the positive control without sample followed the same method using XNG and HPMC-based SDs. The same procedure served as the negative control without both sample application (XNG and HPMC SD) and blood input. Thrombose percentage quantification for each sample occurred through application of Equation 9 in the analyses.

$$Thrombose\ (\%) = \frac{mass\ of\ test\ sample - mass\ of\ (-)control}{mass\ of\ (+)\ control - mass\ of\ (-)control} \times 100 \tag{13}$$

S3.3.2. Haemolytic potential

The measure termed "Hemolysis" indicates the extent to which a particular sample induces blood breakdown after touching blood samples during a defined time interval. Hemolytic potential determination procedures and protocols were explained in detail through an American Society for Testing and Materials (ASTM) publication.

The experimental solution of 500 mg received PBS hydration at 37 °C ± 0.5 for an incubation time of 24 h. Washing the sample took place with PBS until it was completely clean. The mixture of sample and defined citrate blood PBS solution was kept at 37 °C with a temperature range between ±0.5 for three hours.

The mixture was centrifuged at 104 rpm for 15 minutes in order to obtain the supernatant following the incubation period. An OPD measurement occurred using a UV/Vis spectrophotometer at 540 nm wavelength for the supernatant solution. The method used the same procedure to analyze both

citrate blood and distilled water serving as the positive controls. The researchers used PBS alone instead of distilled water to serve as the negative control. The glacial Acid Measured through Equation 10 yielded the results as a percentage.

$$Haemolytic\ index\ (\%) = \frac{OPD\ of\ test\ sample - OPD\ of\ (-)control}{OPD\ of\ (+)\ control - OPD\ of\ (-)control} \times 100 \tag{14}$$

S3.4. In vivo X-ray study of SDs

X-ray testing of OFL SDs made from combination XNG and HPMC was performed in vivo through O-X and O-H formulations. SD tablet activity was investigated in a dog animal model through the gastrointestinal system by examining compressed tablets of SDs

S3.4.1. Preparation of tablets

A direct compression method was used to produce SD tablets from X-ray structure prepared by XNG and HPMC. A direct compression method was employed to prepare tablets containing barium sulphate (BaSO4) instead of 25% SD powder according to the components mentioned in Table 2. BaSO4 served as a contrast agent in tablet formulation because it improves X-ray image visibility (29, 30).

The investigation relied on following parameters from Table 2 to maintain consistency between pre- and post-compression testing of the tablets and avoid incorrect study interpretations.

S3.4.2. Study design

An in vivo X-ray research used four healthy stray dogs between 16 and 18 kg in weight.

The University of Sargodha's Ethical Committee approved this protocol through a letter which they issued on December 2, 2019 as number SU/ORIC/2/21-I. SU/ORIC/2/21-I dated December 2, 2019. The NIH Publications No. 8023 from 1978 underwent amendments to form the foundation for designing this study according to the National Institute of Health's Guidelines for the Care and Use of Laboratory Animals.

Each animal received fasting treatment lasting through an entire night prior to the experiment day. The experimental animals received formulated OFL SD tablets through the gavage tube along with water using XNG (A-X) or HPMC (A-H).

The researcher used X-ray radiography of the animal's stomach to establish that both the stomach was empty and the tablet position before and directly following administration. The trial period restricted all food intake for the animals while they received unaltered tap water in unlimited quantities. A Toshiba Corporation Japanese X-ray equipment with Beam Limiting Device TF-6TL-6 took images of animal stomachs at specified intervals at 70 Kv with 400 mAs.

S3.5. In vitro dissolution and release kinetics

In vitro, the dissolution of compressed tablets of standard OFL and developed SDs (O-H and O-X) was carried out in phosphate buffer (pH 6.8) at 37 ± 0.5 °C for 8 h. The dissolution data in the form of cumulative drug release is plotted against time and provided in Fig. 4a. The results revealed that the maximum release achieved in the case of standard OFL was only 59.2% after the 8th h of the dissolution study. Comparatively, the SDs (O-H and O-X) showed higher dissolution, i.e., 97.88 and 82.34%, respectively, within the same time period. The enhanced dissolution of developed SDs may be due to the hydrophilic nature of HPMC and XNG, as these may expedite the rapid movement of water into the SD matrix. This process led to hydration while speeding up the drug release from the SDs. The reduced particle size led to an enlargement of surface area for dissolution thus promoting the initial drug release from SDs. The drug release rate enhancement can be attributed to three characteristics of polymers (HPMC and XNG) in formulated SDs: increased surface area, non-crystalline form and hydrophilic nature. Similar release kinetic for OFL, atorvastatin calcium/ezetimibe and itraconazole have already been reported.

Different kinetic models (zero order, first order, Higuchi, Hixson-Crowell and Korsmeyer-Peppas) were utilized to fit the dissolution data, to determine release kinetics of the drug from tablets of standard OFL and formulated SDs (O-H and O-X). It was established from drug release kinetic parameters (Table 3.17) that the release of the drug from O-H followed first-order kinetics and from O-X followed the Korsmeyer-Peppas model. The most suitable models for drug release from O-H, O-X and OFL are depicted in Fig. 4(b-d). Moreover, values of n (Table S11) showed that the drug was released by diffusion process from O-H, while the drug released from standard OFL and O-X followed both erosion and diffusion mechanisms.

S3.6. Haemocompatibility studies

The prepared SDs went through a thrombogenicity analysis by measuring the clot weight formed as a result of testing material and blood interactions against the control reference. Results are displayed in Table S5 and showed that the thrombose (%) values of O-H (HPMC-based SD) and O-X (XNG-based SD) were 78.49 ± 3.77% and 73.91 ± 4.19%, respectively. It was also observed that the weight of the blood clots formed with the prepared SDs was less than the positive control. Therefore, these results indicated that the SDs prepared with HPMC and XNG were non-thrombogenic in nature.

The haemolytic index of O-H and O-X was determined as 2.53 ± 1.09% and 1.46 ± 0.69%, respectively. The ISO document 10993-32002 sets safety standards that require the hemolytic index to be below 5% to classify a material as safe for biomedical use. (ISO, 10993-4:2017, TC 194). In the case of both SD formulations, the haemolytic index was less than 5% and comparable to the works of the peers. Hence, SDs prepared from HPMC and XNG are considered haemocompatible which lead to their suitability for biomedical applications.

S3.7. In vivo X-ray study

The behaviour of XNG-based SD (O-X) was detected during transit through the GIT of the dog, and the results are depicted in Fig. 6a. The radiographic image taken after 1 h showed the presence of the tablet inside the animal stomach. The brightness of the tablet with sharp edge indicated the intact nature of the tablet without any breakage or damage to the tablet. After 2-6 h, the tablet moved through different parts of the small intestine. With the passage of time, the tablet moved through different parts of the gastrointestinal tract. The image captured at 8 h showed that the tablet in the colon area while showing reduced size due to its degradation process. The complete disintegration of the tablet was confirmed by the disappearance of the tablet after a 10-hour time period.

Fig. 6b expressed the *in vivo* fate of the SD prepared from HPMC. The tablet can be seen during the 7-hour study period and appeared at different positions of the GIT, which indicated that the tablet had been transited without significant damage. The complete disintegration of the tablet was confirmed by its disappearance after 9 hours. X-ray study of the tablet prepared from XNG and

HPMC-based SD confirmed the prolonged stay in the GIT with slow degradation of the formulation. Hence, these formulations sustained the drug release at least for 9 h.

S3.8. In vitro antibacterial study

In vitro, the antibacterial study of the SD samples was determined against *E. coli* and *S. aureus* through the disc diffusion method and compared with that of the standard drug. The results of the antimicrobial studies are depicted in Tables S13 and S14. A comparison of the study is presented in Figures 14 and 15, which designated that the zone of inhibition of SD samples was comparable to the standard drug (positive control). The zone of inhibition of standard OFL against *E. coli* was noted as 15.5 ± 0.51 mm. At the same time, the zone of inhibition exhibited by O-H and O-X against *E.* Therefore, the values indicated that the prepared SDs showed significant antimicrobial activities against the Gram-negative bacteria and were comparable to the standard. Similarly, the zone of inhibition of SDs prepared with the help of XNG against *S. aureus* was measured as 15.3 ± 1.89 and 17.1 ± 1.32 mm for O-H and O-X, respectively (Figure 15). At the same time, the zone of inhibition of standard OFL against *S. aureus* was recorded as 18.3 ± 0.78 mm. These values are comparable to the standard values (Fig. S2a and S2b). Moreover, there was no antimicrobial activity in the negative control. Therefore, it can be concluded that the prepared SD formulations have demonstrated important antibacterial effects against Gram-negative and Gram-positive bacterial strains.

Hydrophilic polysaccharides (HPMC and XNG) effectively enhanced the solubility of ofloxacin by preparing SD formulations *via* a spray drying technique. The enhancement in solubility may be attributed to three factors, i.e., phase transition (crystalline to amorphous) of drugs, reduction in particle size and presence of hydrophilic moiety. No chemical association was found between the drug and polymeric carriers; however, the presence of a hydrogen-bonded -OH group between the drug and carriers was confirmed. HPMC and XNG-based solid dispersions showed good flow properties, and their compressed tablets exhibited better mechanical strength and equivalent distribution of drugs among SDs. The enhanced dissolution profiles were observed in the case of all fabricated SDs as compared to the standard drugs. Enhanced pharmacokinetic parameters of SDs indicated their enhanced oral bioavailability, which may attributed to increased solubility of SDs. The enhanced half-life values indicated that SDs would have the potential to attain a daily dose and improve patient compliance with drugs. The developed SDs are haemocompatible and

can be utilized for biomedical applications. *In vivo, an* X-ray study indicated the prolonged stay of tablets in the GIT with slow degradation of the formulations. *An in vitro* antibacterial study predicted that prepared SDs exhibited significant antibacterial activities against Gram-positive and Gram-negative bacteria, comparable with standard drugs.

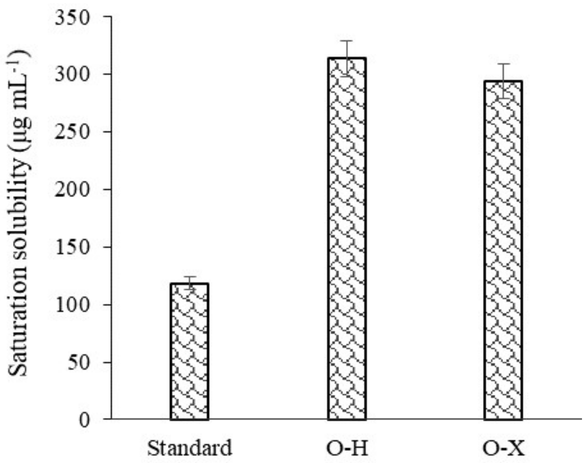

Fig. S1. Saturation solubility of formulated SDs (O-H, O-X) and standard (OFL), conferring

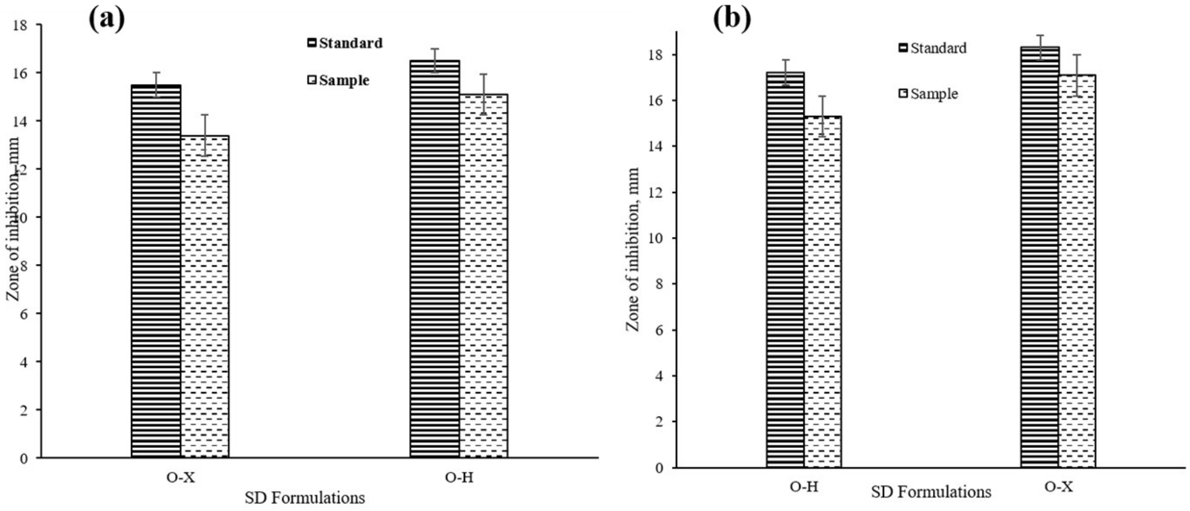

Fig. S2. (a) Zone of inhibition of SD formulations and standard drug against *E. coli*.and (b) zone of inhibition of SD formulations and standard drug against *S. aureus*.

Table S6. Amount of SDs administered to rabbits

Formulation	OFL concentration (mg)	Equivalent amount of SD (mg)
OFL	30	-
O-H	-	90
O-X	-	90

Table S7. Pharmacokinetic parameters of O-H, O-X and standard (OFL) after a single oral dose

Pharmacokinetics parameter	O-H	O-X	Standard (OFL)
C_{max} (μg mL^{-1})	4.33 ± 0.21	4.12 ± 0.33	1.8 ± 0.61
t_{max} (h)	3.0	4.0	2.0
$t_{1/2}$ (h)	8.0 ± 0.25	8.0 ± 0.31	5.0 ± 0.71
$AUC_{0-\infty}$ (h μg L^{-1})	26.88 ± 3.81	25.57 ± 4.43	11.12 ± 2.53
Vd (L kg^{-1})	8.98 ± 0.22	7.85 ± 0.31	1.15 ± 0.41
Cl (L h^{-1})	0.917 ± 0.014	0.98 ± 0.031	1.49 ± 0.055

Table S8. Haemocompatibility studies of SDs based on HPMC and XNG

Formulations (SDs)	Thrombose (%)	Haemolytic index (%)
O-H	78.49 ± 3.77	2.53 ± 1.09
O-X	73.91 ± 4.19	1.46 ± 0.69

Table S9. Saturation solubility and drug contents of OFL, O-H and O-X

Formulation code	Saturation solubility (μg mL^{-1})	Drug contents (%)
OFL	118.31 ± 0.61	-
O-H	313.21 ± 0.73	99.26 ± 0.63

O-X	293.64 ± 0.95	98.44 ± 0.54

Table S10. Stability study of SDs in accordance with ICH guidelines

SD formulations	Drug content (%)		
	2 months*	4 months**	6 months***
O-H	99.26 ± 0.63	99.13 ± 1.43	99.06 ± 1.33
O-X	98.44 ± 0.54	98.24 ± 1.33	98.05 ± 1.21

* Average of daily analysis for 2 months

** Average of analysis after 15 days for the next 2 months

*** Average of analysis after 30 days for the next 2 months

Table S11. Post-compression parameters of compressed SD tablets

Formulation code	Hardness (kg/cm^2)	Thickness (mm)	Friability (%)	Weight variation (%)	Content uniformity (%)
O-H	2.83 ± 0.24	2.31 ± 0.42	0.83 ± 0.11	2.63 ± 0.43	99.06 ± 0.34
O-X	2.71 ± 0.44	2.44 ± 0.71	0.71 ± 0.24	2.73 ± 0.45	98.12 ± 0.44

Table S12. Release kinetic parameters of O-H, O-X and standard (OFL)

Kinetic model	Parameters	Formulations		
		O-H	O-X	Standard (OFL)
Zero-order	$\mathbf{K_0}$ (% h^{-1})	19.92	19.53	11.70
	$\mathbf{R^2}$	0.8303	0.9679	0.8813
First order	$\mathbf{K_1}$ (% h^{-1})	0.267	0.176	0.0719
	$\mathbf{R^2}$	0.998	0.9958	0.9276
Higuchi	$\mathbf{K_H}$ (% $h^{-1/2}$)	45.95	41.95	26.335
	$\mathbf{R^2}$	0.976	0.987	0.985
Hixon-Crowell	$\mathbf{K_{HC}}$ (% h^{-1})	0.628	0.482	0.227

	**R^2**	0.956	0.996	0.913
Korsmeyer-Peppas	**K_{KP}** (% h^{-n})	0.471	0.562	0.549
	**R^2**	0.9926	0.9984	0.9833
	**N**	0.310	0.802	0.501

Table S13. Zone of inhibition of HPMC-based SD formulation and standard drug against *E. coli* and *S. aureus*

Formulation	Zone of inhibition (mm)	
	E. coli	*S. aureus*
Standard	16.5 ± 0.22	17.2 ± 1.43
O-H	15.1 ± 0.62	15.3 ± 1.89

Table S14. Zone of inhibition of XNG-based SD formulation and standard drug against *E. coli* and *S. aureus*

Formulation	Zone of inhibition (mm)	
	E. coli	*S. aureus*
Standard	15.5 ± 0.51	18.3 ± 0.78
O-X	13.4 ± 0.73	17.1 ± 1.32
